# The Protective Effects of Silk Sericin Against Retinal Oxidative Stress: *In Vitro* and *In Vivo* Assays with a Fluorometric Nitroxide Molecular Probe

**DOI:** 10.3390/molecules30244707

**Published:** 2025-12-09

**Authors:** Cassie L. Rayner, Shuko Suzuki, Traian V. Chirila, Nigel L. Barnett

**Affiliations:** 1 Clem Jones Centre for Regenerative Medicine, Faculty of Health Sciences & Medicine, Bond University, Gold Coast, QLD 4229, Australia; crayner@bond.edu.au (C.L.R.); nbarnett@bond.edu.au (N.L.B.); 2 Queensland Eye Institute, Woolloongabba, QLD 4102, Australia; shuko.suzuki@qei.org.au; 3 Australian Institute of Bioengineering & Nanotechnology (AIBN), University of Queensland, St Lucia, QLD 4072, Australia; 4 Faculty of Medicine, George E. Palade University of Medicine, Pharmacy, Science and Technology, 540139 Târgu Mures, Romania

**Keywords:** *Bombyx mori* silkworm, silk sericin, oxidative stress, antioxidants, profluorescent nitroxide, retinal photoreceptor cells, ischemia, reperfusion

## Abstract

Sericin is a major polypeptidic constituent of the silk in the cocoons produced by the *Bombyx mori* silkworm. Certain fractions isolated from sericin exhibited antioxidant properties in a variety of reported experimental settings. In a previous study, we found that only the non-protein fraction, extracted from crude sericin, displayed antioxidative activity in cultures of murine retinal photoreceptor cells (661W), a cell line that is highly sensitive to oxidative stress associated with diseases of the retina. In the same assay, the protein fraction (purified sericin) did not show any such activity. To check these findings, in the present study, two additional different assays were employed: an *in vitro* assay based on the dose-dependent mitigating effects exerted by each sericin fraction on the activity of antimycin A in cultures of 661W cells and an *in vivo* assay based on an animal (rat) model of retinal ischemia/reperfusion injury. In both assays, nitroxide was appended as a fluorometric molecular probe, and fluorescent intensity was monitored by either flow cytometry (*in vitro*) or the Micron IV retinal imaging system (*in vivo*). The *in vitro* assay indicated unequivocally antioxidative capacity for the non-protein fraction and a lack of it for the purified sericin. The *in vivo* assay indicated that each fraction was able to act as an antioxidant. We hypothesized that the ability of purified sericin to display antioxidative activity *in vivo*, but not *in vitro*, was the result of the metabolic degradation of sericin, a process that delivered serine, an amino acid with known antioxidant properties. However, this hypothesis needs experimental confirmation.

## 1. Introduction

The silk thread produced by the domesticated silkworm *Bombyx mori* and other related Lepidopteran larvae contains two proteinaceous compounds, fibroin (~75%) and sericin (~25%) [1]. Employing established methods, each can be conveniently isolated from either cocoons or silk yarn. Although fibroin has been proposed and used for many biomedical applications, this was not the case for sericin. However, owing to research carried out over the last two decades [2,3,4,5,6,7,8], *Bombyx mori* sericin (henceforth BMSS) has found commercial applications as a cytoprotective supplement in cell culture media. Potential applications of BMSS in tissue engineering have also attracted some interest [9,10,11,12,13].

Our present study addresses the antioxidative capacity of sericin in a biological environment, a property of significance that was first demonstrated in 1998 [14]. Whereas antioxidant activity is specific to sericin, but not to fibroin, it is not clear why the former can function as an antioxidant despite many reports asserting such; for an overview of this issue, see reference [15]. In brief, while the protein fractions isolated from sericin displayed antioxidant activity in experimental conditions [14,16,17,18,19,20,21,22,23,24], so did the non-protein (known also as “non-sericin”) fractions accompanying the protein [25,26,27,28,29,30]. To complicate matters, when retinal cells were cultured on fibroin–sericin hydrogel membranes, no antioxidative effects could be detected despite the massive presence of sericin within the solid substrate [31]. In a previous study [15], we found that the non-protein components in BMSS were mainly responsible for the antioxidant activity shown in cultures of retinal photoreceptor cells. In the present study, we employed additional methods to investigate this issue in more depth.

**Figure 1:**
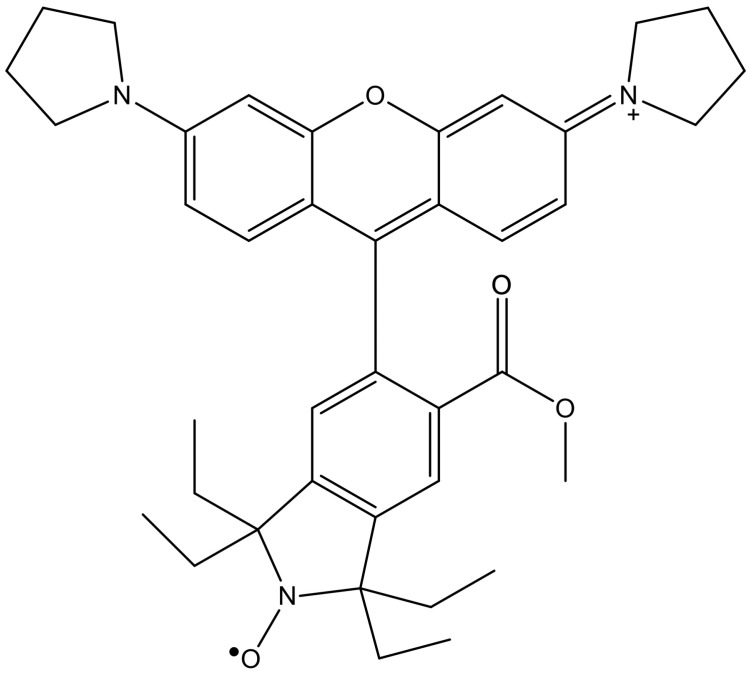
The chemical structure of the molecular probe ME-TRN.

In the new set of experiments, we used a profluorescent nitroxide as a probe to monitor the redox state of the cellular environment. Nitroxides (known also as nitroxyl radicals) are stable free radicals with remarkable physicochemical properties due to their stability. When bonded to fluorophores, the resulting profluorescent nitroxide molecules can function as fluorometric probes for assessing the radical and redox states in a biological system [32,33,34]. We used the methyl ester of tetraethylrhodamine nitroxide (ME-TRN) (Figure 1), a reversibly responsive fluorometric probe, which―like all rhodamine derivatives [35]―is predominantly localized in the mitochondria when administered to cells [34]. Considering that the mitochondrion is one of the main sites for the generation of reactive oxygen species (ROS), this was an important factor when opting to employ ME-TRN as a probe.

Most of the available fluorometric probes act in a one-way direction, i.e., they are turned on and fluoresce in the presence of ROS, but this process cannot be reversed by antioxidants. In contrast, the molecule of ME-TRN changes fluorescence in both directions; it is not fluorescent until is taken up by cells, where it is reduced and turned on. Oxidative stress caused by ROS pushes the probe in the opposite direction and turns off the fluorescence. An antioxidant can either (a) prevent the fluorescence of the probe from being turned off by oxidative stress or (b) turn the fluorescence back on after it has been previously turned off.

For our cell culture experiments, a murine retinal photoreceptor cell line (661W) was selected. These cells express only cone markers and are photosensitive [36]. They have been used previously [15,37,38,39] as suitable models for studying the retinopathies where oxidative stress may play a major role. Oxidative stress is the result of an excessive amount of ROS, i.e., oxidants, produced by cells as compared to the amount of natural endogenous antioxidants. Through certain mechanisms [40,41], an effective antioxidant should be able to prevent the generation of ROS from extracellular or intracellular components (proteins, lipids, nucleic acids).

In the cell culture, we used antimycin A (AMA) to stimulate the production of ROS. AMA is an inhibitor of the mitochondrial electron transfer chain complex III. The inhibition of complex III results in the increased production of ROS in the mitochondria [42,43]; therefore we can say that AMA has a pro-oxidant effect. The fluorescence of the cells was measured by flow cytometry and compared between that of cells treated only with AMA and that of cells treated with both AMA and a putative antioxidant (in this case, a sericin formulation). If the fluorescence is brighter in the presence of antioxidant, it means that the latter has mitigated some of the pro-oxidant effect of AMA.

We also carried out an *in vivo* assay using a rat model of acute retinal ischemia/reperfusion injury. After the ME-TRN probe is injected into the eye of the animal, it is taken up by retinal cells and undergoes reduction (as opposed to oxidation) to become fluorescent. By increasing the intraocular pressure (IOP) to 120 mm Hg, the blood flow in the retina stops and causes ischemia. After an allocated time, the IOP is brought to a normal value, allowing for routine blood flow again (reperfusion). The sudden influx of blood and oxygen induces a massive release of ROS, which decreases the fluorescence of the probe. Using a fluorescence fundus camera, the retina in the live animal can be imaged, and the brightness of fluorescence can be quantified across the retina. If the fluorescence is brighter in an animal that has been treated with a putative antioxidant than in one that has not, this suggests true antioxidant activity.

As described here, the fluorometric method based on the ME-TRN molecular probe can be applied to assess the *in vivo* activity of an antioxidant in real time. This application is significant for the biological environment of the eye, where the redox imbalance is associated with major neurodegenerative disorders such as glaucoma and age-related macular degeneration, both leading to severe visual disabilities.

## 2. Results

### 2.1. Flow Cytometry: In Vitro Quantification of Oxidative Stress

Our previous studies [34,37,44,45] have shown that cultured 661W photoreceptor cells converted the ME-TRN probe from a nonfluorescent oxidized state to a fluorescent reduced state. The administration of the pro-oxidant AMA results in a decline in fluorescence. This unique ability of the ME-TRN molecule to switch states based on the redox state of its environment allowed for a rapid technique by using flow cytometry to quantify *in vitro* the oxidative stress and mitigation provided by a potential antioxidant.

**Figure 2:**
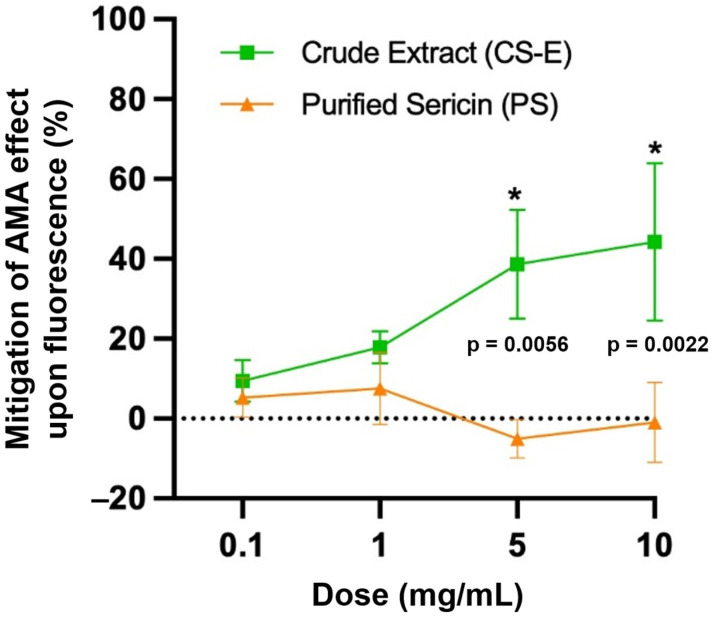
The quantification of oxidative stress and the mitigation of the antimycin (AMA)-induced pro-oxidant effect on the molecular probe’s fluorescence by the sericin samples, CS-E and PS, as putative antioxidants. Assessment was performed by flow cytometry on the cultured 661W photoreceptor cell line. Data are presented as the mean ± s.d. (*n* = 3, * *p* < 0.05), compared with the vehicle control.

Figure 2 shows that the sericin sample CS-E significantly mitigated the pro-oxidant effect of AMA, lessening its suppressive effect on the probe’s fluorescence, at doses of 5 mg/mL (by 38.62 ± 7.86%, *p* = 0.0056) and 10 mg/mL (by 44.29 ± 11.40%, *p* = 0.0022). CS-E is an extract of crude sericin containing predominantly non-protein components. In contrast, purified sericin (sample PS) did not exhibit any mitigating action on AMA’s effect upon fluorescence, thus suggesting that PS may not meet the requirements for an antioxidant in accordance with the testing conditions.

### 2.2. In Vivo Quantification of Fluorescence Intensity in Rat Retina

**Figure 3:**
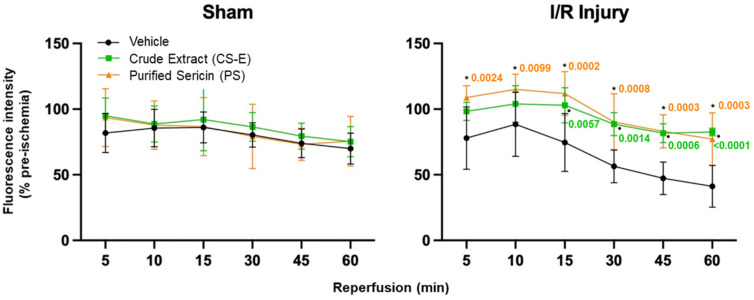
The quantification of ME-TRN fluorescence captured *in vivo* in the rat fundus during 60 min of reperfusion following an induced acute ischemic insult. Data are presented as changes in the fluorescence intensity at each time point as a percentage of the pre-ischemic (time zero) value (mean ± s.d.). In the non-ischemic eyes (sham), the intraperitoneal administration of sericin samples (CS-E, PS) had no effect on retinal fluorescence throughout the treatment compared to the vehicle group (*p* = 0.7204). An I/R injury resulted in a marked decrease in fluorescence intensity over the 60 min of reperfusion (I/R injury, vehicle). Intraperitoneal injections of any of the sericin samples (CS-E or PS), administered one hour before the ischemic insult, mitigated the decrease in retinal fluorescence intensity. Significant improvements were observed at each time point for PS (*, *p* < 0.05) and after 15, 30, 45, and 60 min for CS-E (●, *p* < 0.05), compared with I/R injury vehicle controls at each respective time point, as indicated by the inserted *p*-values.

Through the use of the innovative ME-TRN probe along with the Micron IV imaging system, the real-time, *in vivo* quantification of retinal oxidative status was possible in an animal (rat) model of acute I/R. ME-TRN specifically displays fluorescence changes in both pro-oxidant (I/R) and antioxidant circumstances. I/R injury is a well-established pro-oxidant stimulus *in vivo*, rapidly inducing an excess of ROS. We have previously shown [45] that this probe remains relatively stable *in vivo* over a 120 min treatment period, i.e., 60 min sham ischemia and 60 min sham reperfusion plus imaging, in the non-ischemic control eyes. Here, the apparent decrease in fluorescence from 81.8% to 69.9% over the 60 min sham reperfusion period is small but statistically significant (Figure 3, sham, vehicle, black plot; one-way repeated measures ANOVA (F_(5,25)_ = 17.07, *p* < 0.0001)). The ROS generated during reperfusion leads to a significant time-dependent decrease in retinal fluorescence. In this study, we compared two putative antioxidants isolated from natural sericin in their ability to reduce oxidative stress in the retina subsequent to an I/R injury.

As shown in Figure 3, in the non-ischemic eyes (sham I/R injury), none of the sericin samples (CS-E or PS) had any effect on the fundus fluorescence of ME-TRN throughout the treatment period, when compared to the vehicle control group (F_(2,15)_ = 0.3353, *p* = 0.7204). Based on this, any variation in fluorescence observed between I/R and I/R involving a sericin sample was regarded as being caused by antioxidative activity that was able to diminish the overall production and accumulation of ROS within the retina.

When each of the samples, either CS-E or PS, was administrated one hour prior to the I/R injury, both mitigated the decrease in injury-induced fundus fluorescence to a similar degree (F_(2,15)_ = 12.71, *p* = 0.0006). Their presence blunted significantly the decrease in retinal fluorescence at each time point compared to the injury group, as seen in Figure 3, where the *p*-values are attached to the data points for CS-E (●) and for PS (*), respectively.

## 3. Discussion

Chemically speaking, BMSS is a complex composite of copolypeptides, accompanied by lower amounts of phytochemicals (e.g., terpenoids, phenolics, carotenoids, alkaloids), other proteins (e.g., enzymes, seroins), carbohydrates, and mineral matter. Details about the major protein components in BMSS are still in deliberation; up to 15 polypeptides have been reported so far, with a mass distribution between 20 and 400 kDa [46], while genomic analysis [47,48,49,50] has indicated that BMSS contains at least 6 polypeptides that were all synthesized in silkworms’ middle gland. For our investigation, we regenerated two BMSS fractions from silk cocoons: an extract from the crude sericin (CS-E) expected to contain mainly non-protein components (e.g., antioxidant flavonoids and carotenoids) and a purified sericin fraction (PS) that should be predominantly proteinaceous matter. The electrophoretic analysis of PS, reported in our previous study [15], has shown a diffuse array of polypeptides covering a molecular mass interval from ~5 kDa to ~60 kDa; the contiguous distribution and low molecular mass are a consequence of hydrothermal degradation during purification procedures. Furthermore, the presence of flavonoids in CS-E and their absence in PS were demonstrated by two different chromogenic assays [15].

An *in vivo* model is much more intricate than an *in vitro* cell culture model, and this can affect the activity of any interfering bioactive agents such as antioxidants. There are complex interactions and processes *in vivo* due to a variety of factors including different cell types, the presence of the extracellular matrix (ECM) and many other biomolecules, metabolic activity, blood flow, and immune responses. Most of these factors are able to alter the antioxidative capacity of a substance and cannot be duplicated in a cell culture setting. Importantly, an antioxidant molecule can undergo significant chemical changes due to such *in vivo* metabolic activity. Therefore, *in vivo* testing provides ideal conditions to evaluate the actual activity of an antioxidant in real time. As a corollary, there are agents or drugs that may work *in vivo*, but not *in vitro*, although the opposite situation seems to occur more frequently.

In Table 1, we summarize the results of this study and compare them with those obtained in our previous study [15]. While the CS-E sample behaved as an antioxidant in all tests, it can be seen that the PS sample displayed minor antioxidant activity that was only detectable through a chemical assay [15], but there was no activity when tested in cell culture, in both our previous [15] and present studies (Figure 2). These findings can be rationalized based on the presence of non-protein antioxidant components (flavonoids, etc.) in CS-E and their absence in PS. However, when subjected to *in vivo* testing in our I/R injury animal model, PS emerged as an apparent antioxidant, at least as effective as CS-E (Figure 3). Although we do not have an immediate explanation for such an apparent discrepancy, we surmise that this is another case where the antioxidant effect is manifested *in vivo* but not *in vitro*. Indeed, the limitations of the *in vitro* evaluation of potential antioxidants and the informational value of the *in vivo* models have been previously noted with regard to flavonoids [51,52,53], α-lipoic acid [54], mitochondria-targeted antioxidants [55,56], and resveratrol [57,58].

The *in vivo* antioxidant activity of PS may be related, at least in part, to its composition. Although virtually devoid of chaperone flavonoid molecules [15], the polypeptides in PS can still be associated with unidentified substances that possess antioxidative properties. An understanding of the reasons why these properties are manifested only *in vivo* entails primarily identifying the putative antioxidant molecules presumably residing within PS. Such molecular entities may include either (a) non-protein antioxidant substances that absconded removal during purification or (b) polypeptides able to exhibit antioxidative properties. Outwardly, the alternative (b) would be a rather surprising finding, as the sericin gels *per se* did not show any antioxidant activity as a growth substrate in cell cultures [31]. However, we suggest here another scenario. Sericin, like any other protein, is metabolically degraded in the body, leading to low-molecular-mass peptides and native amino acids. L-serine is, by far, the most abundant amino acid in the composition of sericin [59], and its metabolic release into the tissues has been manifested as an increase in serine levels in experimental animals fed with sericin [60]. In the body, L-serine is further transformed into D-serine through the enzymatic action of serine racemase, further metabolized, and then pivotally involved in a number of pathophysiologic processes [61,62]. Serine also proved to be an effective antioxidant [63], which is the most relevant for our discussion. In our experiments, sericin was injected peritoneally and certainly became exposed to metabolic degradation at various sites in the body of experimental animals, thus leading to the release of serine as a potential antioxidant. The cultured retinal photoreceptor cells are not capable of phagocytosis, and they are unable to recognize sericin as a foreign body and induce its phagosomal degradation *in vitro*. Therefore, our proposed scenario would be valid only for *in vivo* evaluation conditions. Furthermore, it was demonstrated by nuclear magnetic resonance spectrometry [64] that serine hydroxyl linkages are responsible for the structural stability of sericin through their contribution to the formation of β-sheet aggregates. Therefore, the degradation of sericin up to the stage of releasing serine results actually in the structural collapse of its composing polypeptides, a digestion process that requires much more efficient metabolizing conditions than those attainable in a culture of non-phagocytizing retinal photoreceptor cells. As a matter of fact, there are phagocytic cells in the retinal space, namely, retinal pigment epithelial (RPE) cells—some of the most active specialized phagocytes in our body. The role of RPE cells is to digest the mature outer segments shed by the photoreceptor cells in a process of continuous renewal [65,66]. The next experimental step would be to assess the effect of PS in cultures of RPE cells.

Evidence of antioxidative activity is consistent with our hypothesis. However, this hypothesis is rather speculative at this stage and needs future confirmatory experimental support, such as through the quantification of serine levels in the tissues of animals subjected to treatment. Mechanisms based on the enzymatic modulation of the *in vivo* redox equilibrium or other endogenous natural defence strategies with or without the involvement of metabolites [40,67] could also be considered, but we have no data to support such assumptions.

The low-level antioxidant capacity of PS was previously revealed through a chemical test (TEAC assay) [15], a finding that was associated with the inherent differences between a chemical test and evaluation *in vitro* or *in vivo*. For instance, the TEAC assay can only reveal the ability of a substance to engage in certain chemical reactions, regardless of its environment, while the evaluation of the same substance *in vitro* (cell culture) or *in vivo* (animal models, clinical trials) embodies complex processes where a multitude of biological factors play consequential roles. In fact, it was suggested [68] that a substance should not be considered a genuine biological antioxidant based only upon the results of chemical assays.

## 4. Materials and Methods

### 4.1. Materials

Silk cocoons (*B. mori*), supplied by Tajima Shoji Co., Ltd. (Yokohama, Japan), were produced by silkworms that were fed on fresh natural mulberry leaves.

All chemical reagents and dialysis tubes were supplied by MilliporeSigma (St Louis, MO, USA). Minisart^®^ filters (0.7 and 0.22 μm) were supplied by Sartorius Stedim Biotech (Göttingen, Germany). All cell culture reagents and supplements were purchased from Thermo Fisher Scientific (Rockford, IL, USA), except for the fetal bovine serum (FBS) that was supplied by Cytiva (Sydney, Australia) and bovine serum albumin (BSA) supplied by MilliporeSigma. Tetracaine hydrochloride was supplied by Chauvin Pharmaceuticals, Kingston-Upon-Thames, UK. The methyl ester of tetraethylrhodamine nitroxide (ME-TRN) was synthesized in-house [34]. High-purity water or water for injections was used in all experiments.

The line of 661W murine retinal photoreceptor cells originated in Professor Muayyad Al-Ubaidi’s laboratory at University of Oklahoma Health Science Center (Oklahoma City, OK, USA) and was generously provided to us by Dr Krisztina Valter-Kocsi (Australian National University Medical School, Canberra, Australia).

### 4.2. Isolation of BMSS from Silk Cocoons

To isolate BMSS, autoclave extraction was applied according to our previously published protocols [15,69]. In brief, 10 g of cocoon material was placed in 200 mL water and autoclaved at 121 °C for 4 h. Two different samples were then prepared. A *purified* sericin powder (henceforth PS) was obtained by the dialysis of the solution (MMCO of 3.5 kDa) in water, followed by microfiltration. The solution was frozen at −80 °C and concentrated to a powder in a freeze-dryer/vacuum concentrator (Alpha 1–2 LDplus, Martin Chris GmbH, Osterode, Germany). The same procedure was followed, but omitting the dialysis and filtration stages, to produce the *crude* sericin powder (henceforth CS).

### 4.3. Extraction of Non-Sericin Fraction from Crude BMSS

A previously published protocol [15] was followed. In brief, about 2 g of freeze-dried CS powder was extracted in 150 mL methanol by shaking at 240 rpm for 2 days on a shaker (Model 130 Basic, IKA, Staufen, Germany). The resulting liquid phase was successively filtered and microfiltered and then concentrated to about 15 mL in a rotary evaporator (Rotavapor R-215, Büchi Labortechnik AG, Flawil, Switzerland), followed by drying in an oven at 60 °C overnight. The resulting dry sample (henceforth CS-E) was kept in a vacuum oven at 40 °C for 2–3 days under moderate vacuum and stored in a refrigerator until use.

### 4.4. In Vitro Assays

#### 4.4.1. Cell Culture

Initially, the 661W cells were cultured in T75 tissue culture flasks, in Dulbecco modified Eagle’s medium (DMEM) supplemented with 10% fetal bovine serum (FBS), 2 mM L-glutamine, 50 U/mL penicillin, and 50 mg/mL streptomycin. The cells were maintained at 37 °C in an incubator with 5% CO_2_ and harvested upon reaching 80–85% confluence. For experiments, cells were seeded onto 6-well plates at a density of 38,400 cells/well and allowed to proliferate for 24 h in the medium supplemented with 10% FBS. Serum concentration was then reduced to 1% to slow proliferation and promote the normal functionality of cells. The experiments were conducted on the third day after seeding.

#### 4.4.2. Oxidative Stress Probe

A solution in dimethyl sulfoxide (DMSO) of ME-TRN of known concentration was diluted with 1% FBS-supplemented culture medium to a concentration of 100 nM. A total of 2 mL of this solution was added to each well, and wells were maintained for 45 min to allow for the uptake and accumulation of ME-TRN within the cells.

#### 4.4.3. Treatment with Sericin

The PS and CS-E powdered samples were each dissolved in DMEM supplemented with 1% FBS to achieve successive concentrations between 0.1 and 10 mg/mL, and the solutions were maintained for 30 min before use in cell experiments.

#### 4.4.4. Induction of Oxidative Stress with Antimycin A (AMA)

Before the flow cytometry analysis, cells were exposed to AMA (1 μM) and treated with the sericin formulations for 15 min.

#### 4.4.5. Flow Cytometry and Data Analysis

A flow cytometer machine (BD FACSAria Fusion, BD Biosciences, Macquarie Park, Australia) was used to monitor the changes in the fluorescence intensity of ME-TRN due to oxidative stress and the effects of the putative sericin antioxidants (PS, CS-E). Following treatment, the cells were briefly washed in Dulbecco’s phosphate-buffered saline (DPBS) without Ca^2+^ and Mg^2+^ (Gibco Cat. #14190250) and harvested using the TrypLE enzyme. Cell pellets were then suspended in DPBS with Ca^2+^ and Mg^2+^ (Gibco Cat. #14040182) supplemented with 0.1% BSA and 2 mM EDTA. This solution maintains the viability of cells and prevents their clumping during cytometry.

Before running the samples, BD^®^ CS&T calibration beads (BD Biosciences, Macquarie Park, Australia) were used to compensate for channel cross-talk. Complete details on this analysis were given in a previous report [44].

The mean fluorescent intensities were initially expressed as percentages of fluorescence relative to the ME-TRN control. The data presented (mean ± s.e.m.) resulted from 3 replicate measurements and show the percentage mitigation provided by the treatment with sericin formulations, based on the percentage reduction in the fluorescence of AMA-treated cells alone. Statistical analysis was performed using a one-way ANOVA with Dunnett’s post hoc test.

### 4.5. In Vivo Assays

#### 4.5.1. Animals and Treatments

All animal experiments were conducted in accordance with the current regulations and supported by the required ethical approvals, as detailed at the end of this article.

Albino Sprague-Dawley rats (female, ~250 g) were obtained from the Animal Resources Centre (Canning Vale, Perth, Australia) at 8 weeks of age and housed at Bond University, Gold Coast, Australia. They were kept in rooms with controlled temperature (~37 °C) and humidity (60–70%), with *ad libitum* food and water. A cycle comprising 12 h of light and 12 h of dark (with lights turned on at 7 a.m.) was maintained, and illumination was supplied by overhead fluorescent white lights.

The animals were used to study the potential antioxidant effect of the sericin samples. They received an intraperitoneal injection (10 mg/kg body weight) of either vehicle (water for injections) or a sericin sample at a concentration of 10 mg/mL. As the rats weighed about 250 g each, a volume of 250 μL was injected in each animal, one hour before inducing ischemia/reperfusion (I/R) injury. The injuries included either “sham I/R” or “acute I/R” injury. An acute injury results when the IOP is deliberately elevated to cut off all retinal and choroidal circulation, thus causing ischemia. The sham injury is achieved in identical conditions but without elevating the IOP. The generation of reactive oxygen species (ROS) following I/R injury is known to provide *in vivo* pro-oxidant circumstances [70] and was used in this instance to quantify the potential antioxidant effect of sericin.

The animals’ eyes were divided into six treatment groups (*n* = 6 per group), with the right eye of each rat serving as the sham control and the left eye subjected to the experimental I/R injury according to the following distribution: (i) sham I/R injury, vehicle only; (ii) sham I/R injury, sample CS-E; (iii) sham I/R injury, sample PS; (iv) acute I/R injury, vehicle only; (v) acute I/R injury, sample CS-E; (vi) acute I/R injury, sample PS.

#### 4.5.2. Administration of ME-TRN Probe

The ME-TRN oxidative stress probe was administered as previously described [45]. Briefly, animals were anesthetized with an intraperitoneal injection of 66 mg/kg ketamine and 6.6 mg/kg xylazine. Pupils were dilated with topical 1% tropicamide plus 2.5% phenylephrine, and corneas were anesthetized with topical 0.5% tetracaine hydrochloride. ME-TRN (2 µL of a solution of 50 µM in 10% DMSO in water for injections) was injected into the vitreous using a 10 µL Hamilton syringe with a 26-gauge needle, achieving a final concentration of 2 µM. Intravitreal injections were made posterior to the superior limbus at a 45° angle to avoid touching the lens.

The probe was allowed to disperse throughout the vitreous and accumulate in the retina for 30 min before recording pre-ischemic retinal images using a Micron IV Retinal Imaging System for rodent fundus (Phoenix-Micron, Inc., Bend, OR, USA) equipped with rhodamine filters (Ex 556 nm/Em 590 nm). Pre-ischemic fundus images provided baseline intensity measures for comparing the changes in the ME-TRN fluorescence signal induced by superoxide production during reperfusion and assessing the protective effect of sericin sample intervention. To determine whether sericin pre-treatment mitigates the effect of I/R on ROS generation in the retina, fluorescence intensity was measured at 5, 10, 15, 30, 45, and 60 min after the ischemic insult during the reperfusion phase. Data are expressed as fluorescence intensity as a percentage of the values recorded prior to the insult.

To ensure consistent fundus images before and after the I/R injury, and following the administration of sericin samples, the optic nerve and major blood vessel locations were identified, and pre-ischemic camera and animal stage positioning were fixed. Animals were simply lifted away from the Micron IV camera to allow for correct repositioning after treatment. Bright-field images (with consistent illumination settings) were captured at each time point to verify fundus location before capturing the fluorescence images. For each animal, consistent levels of the excitation illumination and gain settings were used to obtain the fluorescence intensity images, and such levels were maintained throughout the treatment.

#### 4.5.3. Acute Retinal Ischemia by Elevation of Intraocular Pressure

Unilateral ischemia was induced as previously described [44,71,72]. Briefly, the anesthetized animals were immobilized by positioning their front teeth over a horizontal bar and securing the skull with adjustable rods inserted into the bony ear canals. The anterior chamber was cannulated with a 30-gauge needle attached to a reservoir containing sodium chloride solution (0.9%). The needle was inserted into the anterior chamber parallel to the iris plane at the 12 o’clock position. IOP was increased to 120 mm Hg by lifting the reservoir to a height of 163 cm. The blanching of the iris and interruption of the retinal circulation confirmed ocular ischemia. Corneal hydration was maintained throughout with GenTeal gel (Novartis, Alcon Laboratories Australia Pty Ltd., Macquarie Park, Australia). After 60 min of maintaining the elevated IOP, the cannula was removed to allow for reperfusion, thus generating ROS upon the restoration of blood flow.

#### 4.5.4. Image and Data Analysis

**Figure 4:**
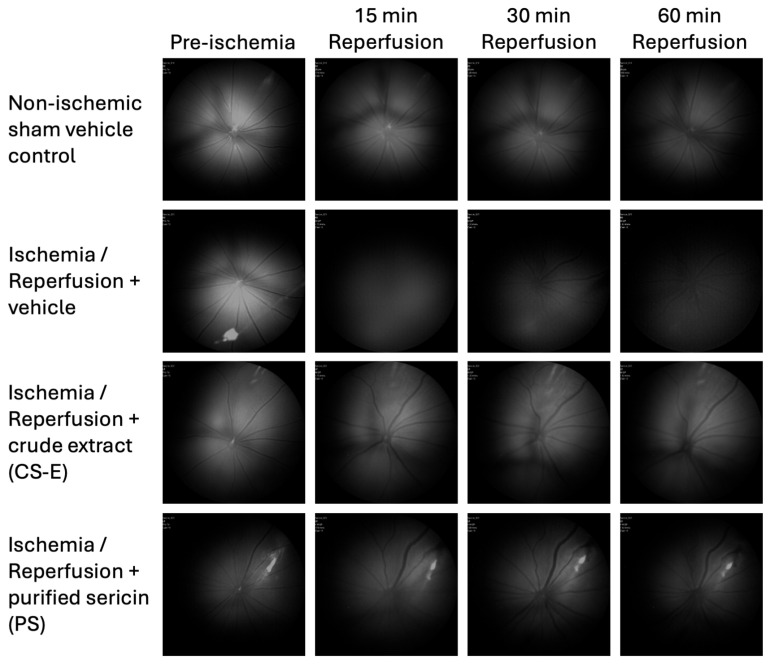
*In vivo* fundus images illustrating the time-course of ME-TRN fluorescence (excitation/emission: 556/590 nm) in rat retinas during reperfusion following acute ischemia and the effect of administering sericin formulations (CS-E or PS). Fluorescence remained stable in the non-ischemic (sham) retinas throughout the 60 min reperfusion period (top row). In contrast, reperfusion after ischemia induced a marked, time-dependent reduction in probe fluorescence, consistent with the generation of ROS (second row). The intraperitoneal administration of sericin samples, either CE-E or PS, mitigated the I/R-induced decline in retinal fluorescence (bottom rows).

Colour fluorescence fundus images were imported into Image J software (version 1.53q, National Institutes of Health, Bethesda, MD, USA) and converted to 8-bit grayscale. These *in vivo* images are shown in Figure 4. In order to provide values of the fluorescence intensity for each time point, the average pixel intensity across the circular fundus image was calculated. To account for possible variability in absolute fundus fluorescence levels between animals, caused by probe diffusion or other confounding factors, data are presented as the change in fluorescence intensity at each time point during reperfusion as a percentage of the corresponding pre-ischemic value (mean ± s.d.). After confirming normal distribution with a Kolmogorov–Smirnov test, statistical comparisons were made using a two-way repeated measures ANOVA with Tukey’s multiple comparison post hoc test. A *p*-value of <0.05 was considered statistically significant.

## 5. Conclusions

Two sericin fractions regenerated from *B. mori* silk cocoons display antioxidant activity in assays involving the use of a nitroxide molecule as a fluorometric probe. The extract of crude sericin, predominantly containing sericin-associated non-protein components, shows antioxidant properties by mitigating the effect of antimycin A on fluorescent intensity in *in vitro* cultures of murine retinal photoreceptor cells. A sample of purified sericin shows no antioxidant activity according to the same assay. Nevertheless, in an *in vivo* rodent model of retinal ischemia/reperfusion injury, the antioxidant properties of both fractions are confirmed. The ability of a purified sericin fraction to display antioxidant activity *in vivo*, but not *in vitro*, can be tentatively explained by the metabolic degradation of sericin *in vivo*, leading to the release of serine, which is the main constitutive amino acid of sericin and a potent antioxidant by itself. At this stage, this is a disputable assertion that needs to be proven by further research.

It is significant to find and investigate natural antioxidants that can efficiently protect retinal photoreceptor cells in order to develop treatments for age-related macular degeneration and other eye diseases that are causally associated with oxidative stress.

## Figures and Tables

**Table 1:** Antioxidant activities of crude sericin extract (CS-E) and purified sericin (PS).

Sample	Antioxidant Activity	Assaying Method	Reference
CS-E	Yes	A	[15]
	Yes	B	This study
	Yes	C	[15]
	Yes	D	This study
PS	No	A	[15]
	No	B	This study
	Minor	C	[15]
	Yes	D	This study

A: Action of an oxidant (H_2_O_2_) on cell viability in cultures of retinal photoreceptor cells. B: Antimycin-induced production of ROS in cultures of retinal photoreceptor cells. C: Trolox equivalent antioxidant capacity (TEAC assay). D: Ischemia/perfusion injury rat model.

## Data Availability

The original contributions presented in this study are included in the article. Further inquiries can be directed to the corresponding author.

## Abbreviations

AMA	Antimycin A
BSA	Bovine serum albumin
BMSS	*Bombyx mori* silk sericin
CS	Crude sericin
CS-E	Methanolic extract from crude sericin (dry)
DPBS	Dulbecco’s phosphate-buffered solution
DMEM	Dulbecco’s modified Eagle’s medium
DMSO	Dimethyl sulfoxide
EDTA	Ethylenediaminetetraacetic acid
FBS	Fetal bovine serum
IOP	Intraocular pressure
I/R	Ischemia/reperfusion
ME-TRN	Methyl ester, tetraethylrhodamine nitroxide
MMCO	Molecular mass cut-off
PS	Purified sericin
ROS	Reactive oxygen species
RPE	Retinal pigment epithelium
TEAC	Trolox equivalent antioxidant capacity
